# Impact of fluoride varnish and oral health education on caries increment and dental plaque microbiota among children with early mixed dentition: a three-armed 24-month randomized controlled trial

**DOI:** 10.1128/spectrum.00902-25

**Published:** 2025-07-28

**Authors:** Xinyi Zeng, Hai Ming Wong, Qing Zhou, Juan Liu, Ni Zhou

**Affiliations:** 1 Department of Pediatric Dentistry, School and Hospital of Stomatology, Kunming Medical University, Kunming, Yunnan, China; 2 Anning Maternity and Child Care Hospital, Anning City, Yunnan, China; 3 Division of Paediatric Dentistry and Orthodontics, Faculty of Dentistry, The University of Hong Kong, Hong Kong SAR, China; University of the Pacific, Arthur A. Dugoni School of Dentistry, San Francisco, California, USA

**Keywords:** fluoride, health education, caries, mixed dentition, oral microbiota

## Abstract

Dental caries is the most prevalent oral disease globally. A 24-month randomized controlled trial was conducted among 236 children aged between 7 and 8 years (ChiCTR2400084030). They were randomly allocated into three groups and received the following biannual interventions: Group 1: topical fluoride varnish (TFV) using 5% sodium fluoride; Group 2: oral health education (OHE); and Group 3: TFV + OHE. The interventions lasted for 12 months, and a final examination was conducted after 24 months to assess whether the desired effects sustained after the cessation of interventions. After 12-month interventions, when demographic and socioeconomic factors were adjusted, children in Group 1 were more likely to have new caries on their permanent first molars when compared to Group 2 (odds ratio [OR] = 2.86, 95% confidence interval [CI] 1.33, 6.14) or Group 3 (OR = 2.28, 95% CI 1.09, 4.78). There was no significant difference in Shannon diversity index across the three groups. Among the top 10 most abundant genera, *Capnocytophaga*, *Saccaribactetia_(TM7)_[G-1]*, *Selenomonas*, and *Porphyromonas* differed across the three groups after interventions. The 12-month assessment revealed a lower abundance of *Capnocytophaga granulosa* among children who did not receive TFV. Although the interventions might have the potential to alter the composition of supragingival dental plaque, the specific mechanisms and clinical significance require further investigation.

**IMPORTANCE:**

Early mixed dentition is a critical transition period for children. During this phase, newly erupted permanent first molars (PFMs) with deep fissures are vulnerable to tooth decay. Considering increased independence in diet choices and inconsistent toothbrushing practices among these children, structured oral health education (OHE) is recommended to safeguard their oral health status. The main findings of this 24-month randomized controlled trial revealed that fluoride varnish alone is insufficient to prevent caries on PFMs, underscoring the necessity of reinforcing OHE for this age group. Additionally, dynamic shifts in supragingival plaque composition were observed, including *Capnocytophaga*, *Saccaribactetia_(TM7)_[G-1]*, *Selenomonas*, and *Porphyromonas*. Notably, *Capnocytophaga granulosa*, a species previously found among adults with periodontal diseases, was less abundant in children who did not receive fluoride varnish. While these findings highlight the impact of oral health promotion activities on alterations of oral microbiota, further research is needed to clarify the clinical implications of these microbial changes.

**CLINICAL TRIALS:**

The study was registered with the Chinese Clinical Trial Registry (ChiCTR2400084030).

## INTRODUCTION

The mixed dentition stage, characterized by the coexistence of both primary and permanent teeth, occurring typically between the ages of 6 and 12, is a critical transitional period among pediatric dental patients. The health condition during this stage can influence the health status of permanent dentition. However, evidence suggests that children in the mixed dentition stage are likely to encounter dental caries, malocclusion, gingivitis, and extrinsic black tooth stains (EBS) (1–3). A recent study compared the prevalence of dental caries and EBS among children in various dental stages, demonstrating that children in the mixed dentition stage exhibited a higher EBS prevalence than children in the primary dentition and a higher caries prevalence than their peers in the permanent stage (3). Dental caries, a multi-factorial disease, affects over one-third of the global population (4). Extensive caries could lead to premature loss of primary teeth. During the early mixed dentition stage, early loss of deciduous teeth can potentially disrupt the eruption pattern of the succeeding permanent teeth and cause malocclusion (2). Therefore, initiating oral health promotion activities among children in the early mixed dentition stage is crucial to safeguard their oral health.

Topical fluoride varnish (TFV) with 5% sodium fluoride (NaF) at least twice per year has been recommended by the American Academy of Pediatric Dentistry for caries prevention among pediatric dental patients (5). A 24-month randomized controlled trial involving 504 preschool children (6) showed that children in the TFV group had a lower average decayed, missing due to caries, and filled primary tooth (dmfs) score and a higher caries-free rate than their peers who did not receive TFV. Meanwhile, the efficacy of TFV in preventing caries of permanent first molars (PFMs) was supported by another 24-month trial involving 5,005 children with early mixed dentition (7). However, a triple-blind, two-armed, randomized controlled trial conducted in rural areas indicated that no statistically significant inter-group differences in caries status were found between children who received TFV or placebo interventions (8). Moreover, a 24-month randomized controlled trial suggested that if oral health education (OHE) was provided, the biannual application of TFV (5% NaF) did not show additional effects on caries prevention among young children (9). Similar findings were also reported in another trial involving 801 toddlers (10).

Dental caries is associated with children’s toothbrushing habits, dental visit experience, and dietary habits. A 24-month randomized controlled trial (11) conducted among preschool children with special healthcare needs demonstrated that children’s oral hygiene status, toothbrushing performance, and dental attendance behaviors were significantly improved after using OHE interventions. A systematic review also demonstrated that after OHE intervention, children exhibited reduced gingival bleeding scores, more positive attitudes toward dental care, increased toothbrushing frequency, extended toothbrushing duration, and reduced snacking frequencies (12). A meta-analysis further demonstrated that OHE could have a positive influence on dental attendance, dental attitudes, and oral hygiene practices among children (13). Hoeft et al. (14) also reported significant improvements in oral hygiene knowledge among low-income parents, as well as their children’s toothbrushing behaviors, after OHE interventions. Moreover, these changes were maintained three months after the cessation of interventions. Given the evidence supporting OHE’s effectiveness in enhancing the audience’s dental knowledge, attitudes, and oral health-related habits, as well as its cost-effectiveness, OHE might be a promising strategy for oral health promotion in areas with insufficient dental care resources.

Although several studies have investigated the effectiveness of OHE and TFV in caries prevention among preschool children, few studies have investigated the long-term effects of OHE and/or TFV in caries prevention and oral microbiota alterations among children with mixed dentition. Considering that mixed dentition is a critical transitional period for children, oral health promotion in this stage is of utmost importance. Therefore, a 24-month randomized controlled trial was conducted to investigate the impact of TFV and OHE on caries prevention and dynamic changes of oral microbiota among children with early mixed dentition.

## MATERIALS AND METHODS

### Study design

This study was designed as a 24-month, single-blind, three-armed, randomized controlled trial. Ethical approval was granted by the Ethics Committee of the Affiliated Stomatological Hospital of Kunming Medical University (Ref No. KYKQ2021MEC087). Information sheets illustrating the purpose and the process of the project were delivered to parents, and written consent forms were signed by parents who were willing to let their children participate in the study. The study was registered with the Chinese Clinical Trial Registry (ChiCTR2400084030). This trial adhered to the Consolidated Standards of Reporting Trials (CONSORT)-Outcome 2022 Extension (15). The microbiology sequences have been deposited in the SRA database (PRJNA1237516).

### Participant recruitment

Grade 2 students were recruited from a local primary school in Chenggong District with the following inclusion criteria: (i) aged between 7 and 8 years, (ii) in mixed dentition, (iii) without developmental delay or disabilities, and (iv) parents agreed to let their children participate in the study. Children with any of the following conditions were excluded: (i) dental fluorosis or enamel hypoplasia; (ii) special education needs or long-term systematic diseases; (iii) dental age not matched with chronological age; (iv) severe dental anxiety, unable to cooperate with routine dental examination; (v) allergic history to composition of fluoride varnish; and (vi) receiving regular topical fluoride applications at dental hospitals or clinics.

### Sample size calculation

The sample size was determined using PASS 15.0 software. Based upon the previous studies (9, 11, 16), the mean differences in decayed, missing due to caries, and filled teeth for children receiving TFV, OHE, and TFV + OHE were estimated to be 0.50, 0.20, and 0.87, respectively. Given a standard deviation of 0.27, a power of 90%, significance level of 0.05, and an allocation ratio of 1:1:1, a total of 174 children were required. To account for a potential 20% loss of participants during the 24-month follow-up period, at least 220 participants should be initially recruited.

### Randomization, allocation, and blinding

Grade 2 students were recruited from a local primary school that had 10 classes at the Grade 2 level, each with approximately 42 students. Six of these classes were selected using a simple random method and were randomly allocated into three groups. The randomization sequence was generated using Excel software. The allocation sequence was concealed using opaque, sealed envelopes, which were opened after baseline assessment by a single investigator. It was not feasible to blind the participants due to the nature of the TFV and OHE interventions. However, the investigators conducting intra-oral examinations, salivary pH measurement, and statistical analysis were unaware of the group allocations.

### Interventions and follow-ups

Three groups were involved in this study. In Group 1 (TFV), children received biannual applications of 5% NaF varnish (Clinpro White Varnish, 3M ESPE), administered by two trained pediatric dentists following the instructions recommended by the product manual. Children in Group 2 received three OHE leaflets, which highlighted the essential topics related to dental caries prevention, including twice-daily toothbrushing with fluoridated toothpastes, tooth-friendly snacking habits, and regular dental visits (11). Investigators illustrated the key points on the leaflets to the children individually at school, and children were instructed to take the leaflets home to review and practice the illustrated content along with their parents. Moreover, these topics were reviewed during follow-up visits to reinforce children’s understanding of the OHE information. In Group 3 (TFV + OHE), children received both interventions applied to Groups 1 and 2. These interventions lasted for 12 months (Phase I). After 12 months, all interventions were discontinued. A final examination was conducted at the end of the 24th month (Phase II) to evaluate whether the desired effect was maintained after discontinuation of interventions.

### Data collection

#### Intra-oral examination

Three calibrated pediatric dentists performed the intra-oral examinations (kappa values > 0.80). Dental caries experience in mixed dentition was assessed using the number of decayed, missing due to caries, and filled permanent or primary tooth (DMFT/dmft) scores (3). At the 12- and 24-month visits, DMFT/dmft increment (∆ DMFT/dmft) was calculated. As the health status of the PFMs (6s) is crucial for children with mixed dentition, decayed and filled tooth surface (DFS) and the caries incidence of the PFMs were calculated to monitor the caries status on permanent molars (7). Additionally, the severity of carious lesions on each tooth surface was assessed by the International Caries Detection and Assessment System (ICDAS) (17). Codes 3 (“localized enamel breakdown”) to 6 (“extensive distinct cavity with visible dentin”) were recorded, and if a carious lesion progressed to an advanced code, it was defined as “caries progression” (11). The Simplified Debris Index (DI-S) was employed to assess the oral hygiene status among the recruited children, with lower scores indicating better oral hygiene status (18). Caries status was assessed at 12- and 24-month follow-up visits, while children’s oral hygiene status was assessed at 6, 12, and 24 months.

#### Dental plaque collection and analysis

After the intra-oral examination, supragingival dental plaque was collected from the recruited children. Among all the participants, 33 children were randomly selected from the three groups using a simple random sampling method. The dental plaque samples of those children were collected at baseline, 6-, 12-, and 24-month visits. As one child was absent from the 24-month appointment, a total of 131 plaque samples were analyzed. No significant differences were observed in clinical parameters (salivary pH, oral hygiene, and caries status) between children whose dental plaque samples were analyzed and their peers whose samples were not analyzed (Table S1). The methods for plaque collection and sample transportation were consistent with those outlined in our previous publication (19). The process of DNA extraction, polymerase chain reaction amplification, and quality control was elaborated in Supplemental materials. The products were sequenced on the Illumina NovaSeq 6000 platform by Gene Denovo Biotechnology Co., Ltd (Guangzhou, China).

#### Measurement of salivary pH

Salivary pH values of the recruited children were assessed at baseline, 6, 12, and 24 months. Before collecting the saliva samples, participants were advised not to eat or drink for 2 hours and were asked to rinse their mouths and naturally accumulate saliva in their oral cavities. They were guided to tilt their heads down at a 45° angle to facilitate the natural flow of saliva into a sterile container. A calibrated portable Pen-Type pH meter with a flat surface electrode (PH5F, Sanxin, Shanghai, China) was used to measure the pH values of the collected saliva samples (19).

#### Oral health-related behaviors and demographics

Children’s oral health-related behaviors and demographics were reported by parents through a structured questionnaire at baseline. The following items were included: between-meal snacking frequency, toothbrushing frequency, dental visit experience, parents’ education attainment, parents’ occupation status, and monthly household income. The questionnaires were cross-checked before data entry. In cases where items were missing, parents were contacted by a dental assistant to provide the necessary information.

#### Statistical analysis

**Fig 1:**
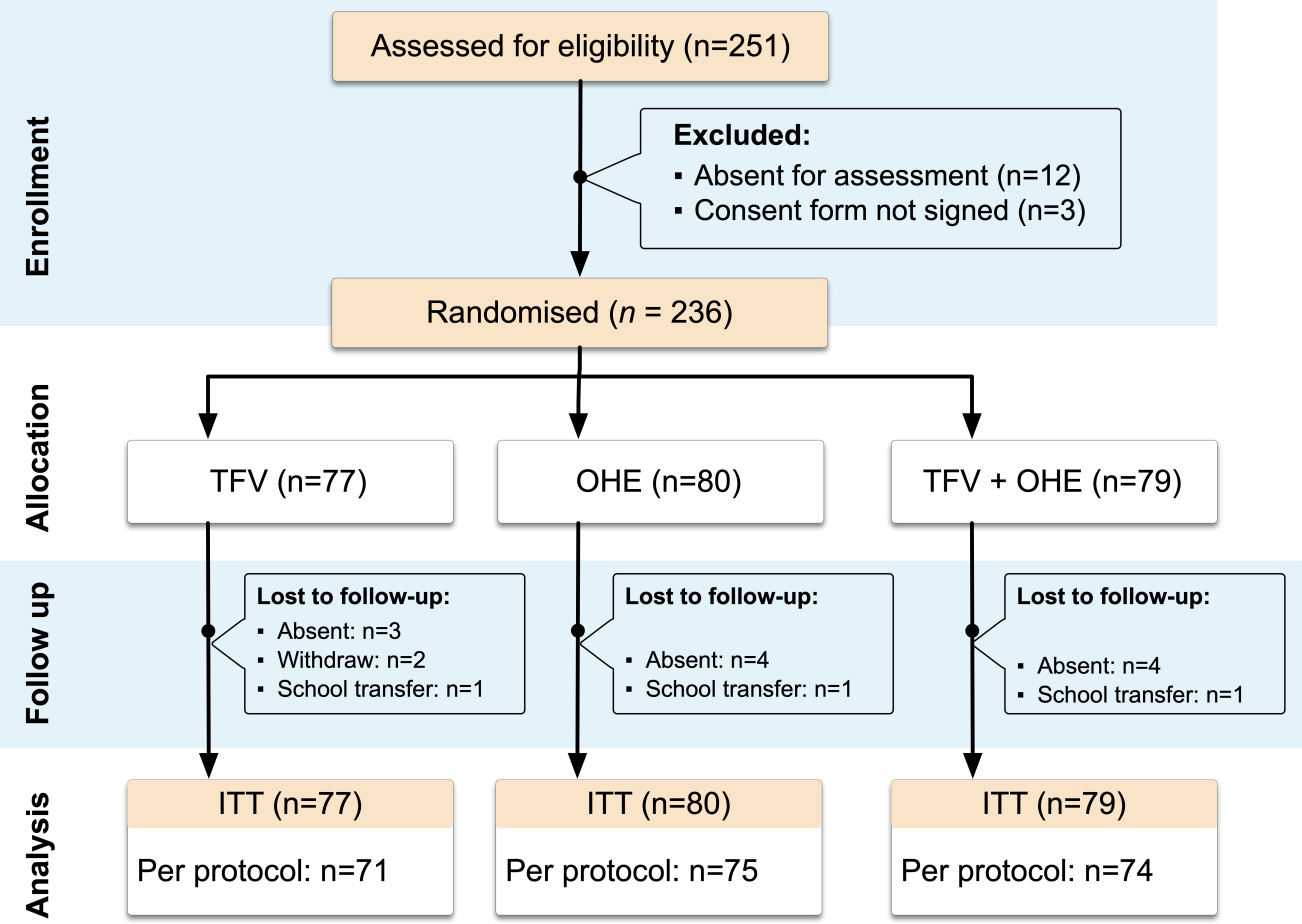
Flowchart of the study process.

Data were analyzed using the IBM SPSS Version 28.0 (IBM Corp, Armonk, New York). Bioinformatic analysis of plaque microbiota was performed using an online platform (http://www.omicsmart.com). Alpha diversity was assessed by Shannon index, while beta diversity was analyzed by principal coordinate analysis (PCoA) at the genus level using Bray-Curtis distance metrics. Regarding the clinical data, both intention-to-treat (ITT) and per-protocol analysis (20) were employed (Fig. 1), yielding similar primary findings. Since the outcomes and *P*-values derived from ITT analysis were slightly more conservative, the results reported are based on ITT analysis. Chi-square tests were conducted to identify differences in caries increment, oral health-related behaviors, and demographics among the three groups. Regarding the continuous variables, one-way analysis of variance was used to compare the differences across the three groups if the data were normally distributed, and Bonferroni *post hoc* tests were performed for multiple comparisons. If the data had a skewed distribution, independent samples Kruskal-Wallis tests were conducted, and the significance values were adjusted by the Bonferroni correction for multiple tests. Factors associated with the caries incidence on PFMs were analyzed using binary logistic regression. Variables such as children’s gender, ethnic groups, parents’ education level, parents’ employment status, and family household income were adjusted in the full models. The significance level was 0.05, and all the comparisons were two-tailed.

## RESULTS

### Participant characteristics and baseline assessment

A total of 251 participants were invited, and 236 children completed the baseline assessment (response rate 94%). During the 24-month follow-up, 16 (6.8%) children dropped out (Fig. 1). No significant differences were observed in the dropout rate across the three groups.

Likewise, there were no significant differences in children’s demographics and their oral health-related behaviors across the three groups. Half of the participants were boys, and approximately a quarter of the participants were children from minority ethnic groups. More than half of the parents held a postgraduate degree, and the majority (91.5%) of the families were dual-income, with both parents working full time. Moreover, over 70% of the families had a monthly income exceeding RMB 10,000 (Table 1).

**TABLE 1:** Characteristics of the recruited children (*n* = 236)^*a*^

Item, % (*n*)	TFV	OHE	TFV + OHE	*P*-value
Gender				
Male, 50.0 (118)	49.4 (38)	50.0 (40)	50.6 (40)	0.987
Female, 50.0 (118)	50.6 (39)	50.0 (40)	49.4 (39)	
Ethnic groups				
Han, 73.7 (174)	68.8 (53)	78.8 (63)	73.4 (58)	0.368
Minorities, 26.3 (62)	31.2 (24)	21.3 (17)	26.6 (21)	
Oral health-related behaviors				
Between-meal snacking				
No, 22.9 (54)	22.1 (17)	23.7 (19)	22.8 (18)	0.969
Yes, 77.1 (182)	77.9 (60)	76.3 (61)	77.2 (61)	
Twice daily toothbrushing				
No, 15.7 (37)	15.6 (12)	15.0 (12)	16.5 (13)	0.968
Yes, 84.3 (199)	84.4 (65)	85.0 (68)	83.5 (66)	
Toothbrushing with fluoridated toothpaste				
Yes, 45.8 (108)	42.8 (33)	51.2 (41)	43.0 (34)	0.091
No, 22.0 (52)	29.9 (23)	21.3 (17)	15.2 (12)	
Unknown or not sure, 32.2 (76)	27.2 (21)	27.5 (22)	41.8 (33)	
Dental visit experience				
No, 12.3 (29)	11.7 (9)	12.5 (10)	12.7 (10)	0.981
Yes, 87.7 (207)	88.3 (68)	87.5 (70)	87.3 (69)	
Family socio-economic status				
Parental education attainment				
Bachelor or below, 41.5 (98)	44.2 (34)	45.0 (36)	35.4 (28)	0.402
Postgraduate, 58.5 (138)	55.8 (43)	55.0 (44)	64.6 (51)	
Both parents employed full-time				
No, 8.5 (20)	5.2 (4)	10.0 (8)	10.1 (8)	0.453
Yes, 91.5 (216)	94.8 (73)	90.0 (72)	89.9 (71)	
Monthly household income				
≤¥10,000, 26.3 (62)	27.3 (21)	27.5 (22)	24.1 (19)	0.859
>¥10,000, 73.7 (174)	72.7 (56)	72.5 (58)	75.9 (60)	

^*a*^ OHE: oral health education; TFV: topical fluoride varnish; data were presented as % (*n*).

The DMFS/dmfs scores among children in the TFV, OHE, and TFV + OHE groups at baseline were 9.31 ± 7.49, 8.10 ± 8.47, and 6.91 ± 6.63, respectively. There were no significant differences in caries experiences, DI-S, and salivary pH values across the three groups (*P* > 0.05, Table 2).

**TABLE 2:** Baseline assessment across the three groups (*n* = 236)^*a*^

Outcomes	Mean (SD)	*P*-value
DMFT/dmft		
TFV	5.01 (3.62)	0.052
OHE	4.05 (3.85)	
TFV + OHE	3.66 (3.14)	
DMFS/dmfs		
TFV	9.31 (7.49)	0.143
OHE	8.10 (8.47)	
TFV + OHE	6.91 (6.63)	
DFS on PFMs		
TFV	0.21 (0.61)	0.628
OHE	0.25 (0.93)	
TFV + OHE	0.14 (0.52)	
DI-S		
TFV	1.13 (0.66)	0.212
OHE	1.11 (0.73)	
TFV + OHE	0.97 (0.49)	
Salivary pH values		
TFV	7.48 (0.47)	0.955
OHE	7.49 (0.41)	
TFV + OHE	7.47 (0.41)	

^*a*^ OHE: oral health education; DI-S: simplified debris index; PFMs: permanent first molars; TFV: topical fluoride varnish.

### Phase I assessment

After 12-month interventions, significant differences were detected in the DFS scores as well as the caries incidence on PFMs across the three groups (*P* < 0.05). The proportion of children with new carious lesions on PFMs was statistically higher in the TFV group (35.1%) than in the OHE group (16.3%). However, there were no statistical differences in the DMFT/dmft increment and caries progression across the three groups (*P* > 0.05). Regarding the DI-S scores, there were no significant differences at the 12-month visit (*P* > 0.05), while statistical differences were found at the 6-month visit (*P* = 0.005). The post hoc tests indicated that children in the TFV + OHE group (1.26 ± 0.60) showed lower DI-S scores than their peers in the TFV group (1.52 ± 0.51) or those children who received OHE intervention (1.50 ± 0.56, *P* < 0.05). The salivary pH levels were significantly higher in the TFV + OHE group (7.27 ± 0.39) and OHE group (7.24 ± 0.39) than the TFV group (7.08 ± 0.39) at 12-month assessment (*P* < 0.05), while no significant difference was found in the salivary pH levels across the three groups at 6-month assessment (*P* > 0.05, Table 3).

**TABLE 3:** Phase I (intervention ongoing) comparisons between the three groups during the first 12 months^*a*^^,^^*b*^

Outcomes	TFV (1)	OHE (2)	TFV + OHE (3)	*P*-value	Comparisons
Full-mouth caries status					
∆ DMFT/dmft	2.10 (2.23)	1.90 (2.00)	2.14 (2.85)	0.651	(1) = (2) = (3)
Caries progression	4.16 (5.20)	3.85 (4.73)	4.84 (6.02)	0.865	(1) = (2) = (3)
Caries on PMFs					
DFS	1.08 (1.70)	0.74 (1.69)	0.68 (1.33)	0.016	(1) > (2), (3)
Caries incidence	35.1 (27)	16.3 (13)	19.0 (15)	0.011	(1) > (2)
DI-S scores					
12 months	1.50 (0.88)	1.49 (0.55)	1.50 (0.62)	0.997	(1) = (2) = (3)
6 months	1.52 (0.51)	1.50 (0.56)	1.26 (0.60)	0.005	(1), (2) > (3)
Salivary pH values					
12 months	7.08 (0.39)	7.24 (0.35)	7.27 (0.39)	0.004	(1) < (2), (3)
6 months	7.37 (0.26)	7.35 (0.24)	7.37 (0.30)	0.855	(1) = (2) = (3)

^*a*^ Categorical data (caries increment) was presented as % (*n*), continuous data were presented as mean (SD).

^*b*^ DI-S: simplified debris index; OHE: oral health education; PFMs: permanent first molars; TFV: topical fluoride varnish.

As significant differences were detected in caries incidence on PFMs after 12 month-interventions, binary logistic regression was employed to estimate the odds ratio (OR) and 95% confidence interval (CI). When demographic and socioeconomic factors were adjusted, children in the TFV group were more likely to have new caries on PFMs when compared to their peers in the OHE group (OR = 2.86, 95% CI 1.33, 6.14) as well as those in the TFV + OHE group (OR = 2.28, 95% CI 1.09, 4.78). However, no significant difference was found between the OHE group and the TFV + OHE group (*P* > 0.05, Table 4).

**TABLE 4:** Caries incidence of PFMs during the intervention period (binary logistic regression)

Interventions	OR	95% CI	*P*-value	Multiple comparisons
Group			0.013	(1) > (2), (3)
TFV (1)	2.86	1.33, 6.14	0.007	
TFV + OHE (3)	1.25	0.55, 2.86	0.590	
OHE (2)^*a*^				

^*a*^ Reference group. CI: confidence interval; OHE: oral health education; OR: odds ratio; PFMs: permanent first molars; TFV: topical fluoride varnish. Note: children’s gender, ethnic groups, parents’ education level, parents’ employment status, and family household income were adjusted.

### Phase II assessment

There was no intervention applied to the participants at Phase II, and a final assessment was conducted at 24 months to determine whether the desired effect was sustained. No significant differences were found in DI-S scores or DMFT/dmft increment across the three groups (*P* > 0.05), while the DFS scores of PMFs differed across the three groups (*P* = 0.032). Children in the TFV group exhibited similar salivary pH levels compared to their peers in the OHE and TFV + OHE groups. However, children in the OHE group demonstrated significantly higher salivary pH values than their counterparts in the TFV + OHE group (Table 5).

**TABLE 5:** Phase II (intervention discontinued) comparisons between the three groups at 24 months^*a*^

Outcomes	TFV (1)	OHE (2)	TFV + OHE (3)	*P*-value
Full-mouth caries				
∆ DMFT/dmft	2.56 (3.13)	1.96 (2.56)	2.05 (2.64)	0.716
Caries progression	5.32 (6.06)	3.59 (4.61)	4.13 (5.17)	0.243
Caries on PFMs				
DFS	1.94 (2.05)	1.35 (2.00)	1.29 (1.80)	0.032
Caries incidence	51.9 (40)	36.3 (29)	39.2 (31)	0.109
DI-S scores				
24 months	1.45 (0.77)	1.35 (0.64)	1.40 (0.74)	0.705
Salivary pH				
24 months	7.13 (0.33)	7.16 (0.33)	7.04 (0.29)	0.036

^*a*^ Categorical data (caries increment) were presented as % (*n*); continuous data were presented as mean (SD). OHE: oral health education; TFV: topical fluoride varnish.

### Changes in oral microbial diversity across the three groups

**Fig 2:**
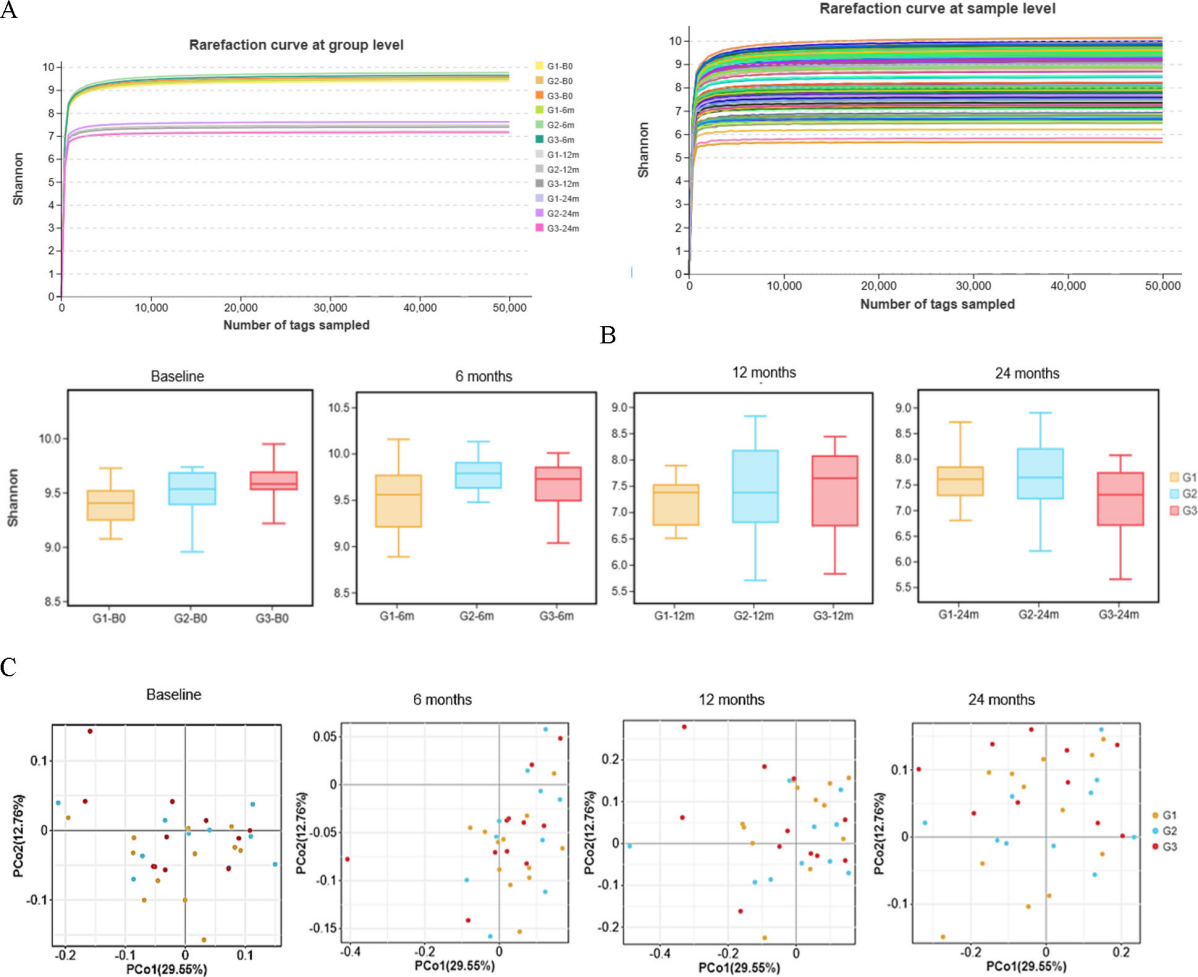
Changes in oral microbial diversity among three groups. (**A**) Rarefaction curves at both the group and sample levels, indicating that a sufficient amount of sequencing data were obtained; (**B**) Boxplots of Shannon index at baseline, 6, 12, and 24 months, showing no significant differences across the three groups (*P* > 0.05, Kruskal-Wallis tests); (**C**) principal coordinate analysis (PCoA) plot using Bray-Curtis distance metric, showing the similarity of the microbial community composition. No statistical difference was found across the three groups (*P* > 0.05, PERMANOVA tests). Note: G1: Group 1 (topical fluoride varnish, TFV); G2: Group 2 (oral health education, OHE); G3: Group 3 (TFV + OHE).

The rarefaction curves demonstrated that the slope of the curves flattens as the effective tags increased, indicating adequate sequencing data (Fig. 2A). The diversity of oral microbial communities was assessed at four time points, including baseline, 6 , 12, and 24 months. No significant differences (*P* > 0.05) were found in Shannon index across the three groups (Fig. 2B). Beta diversity calculated using PCoA revealed that the principal component (PC) one and PC2 accounted for 29.55% and 12.76% of the total variation, respectively. Both the findings at 6 and 12 months revealed a clustering pattern around the positive half-axes of the horizontal coordinate. However, this pattern did not persist after the intervention was discontinued. Additionally, no statistical difference was detected among the three groups by using permutational multivariate analysis of variance (PERMANOVA) analysis (*P* > 0.05, Fig. 2C).

### Dynamic changes in oral microbiota across the three groups

**Fig 3:**
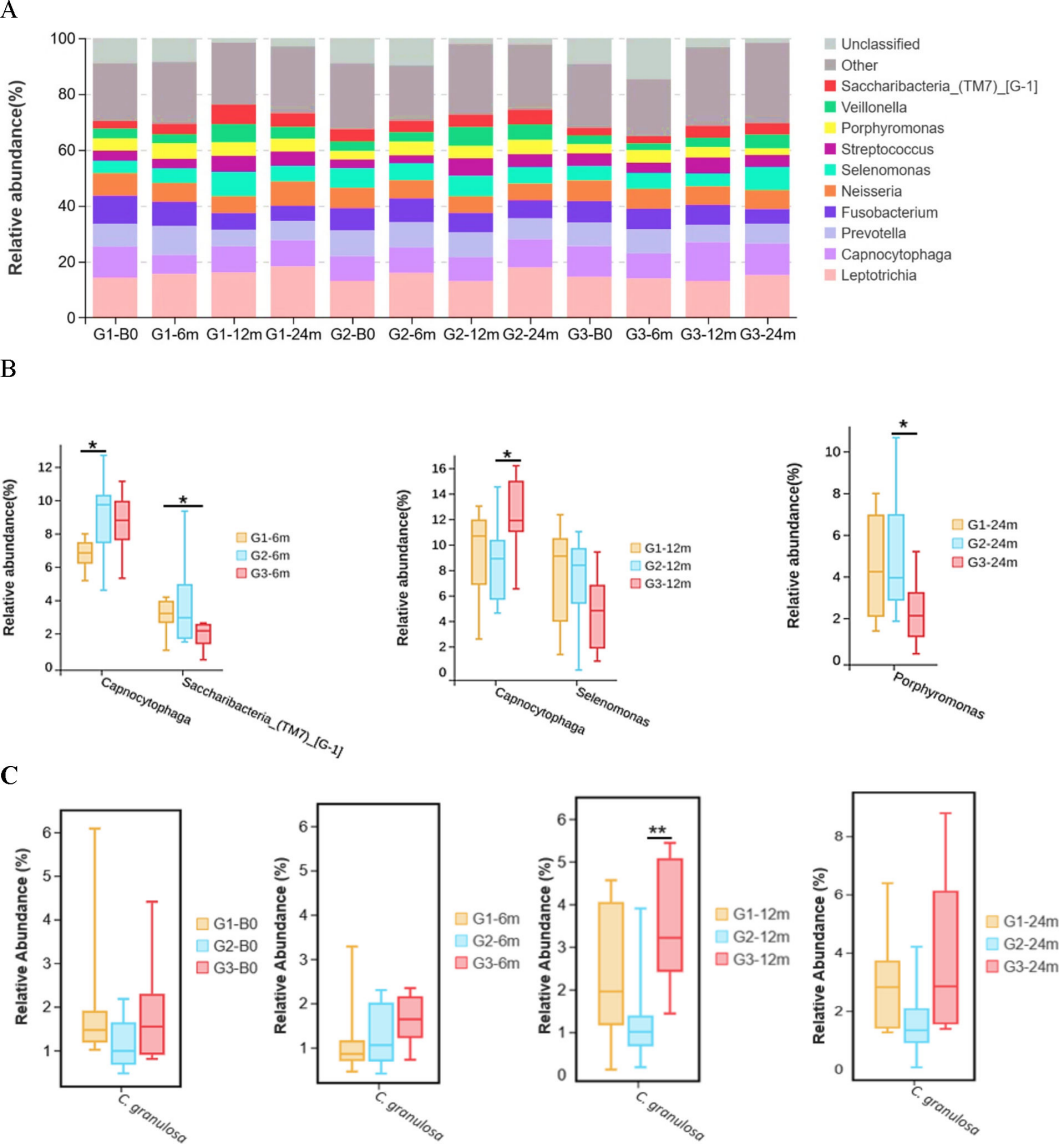
Dynamic changes in oral microbiota during the entire study period. (**A**) Bacterial community composition among three groups at baseline, 6, 12, and 24 months, showing the Top 10 most abundant taxa at genus level; (**B**) boxplots displaying the most abundant genus which differed across the three groups after interventions (Kruskal-Wallis tests). Among the Top 10 most abundant taxa, significant differences were found in the relative abundance of *Capnocytophaga* (*P* = 0.021) and *Saccaribactetia_(TM7)_[G-1]* (*P* = 0.034) at 6 months, *Capnocytophaga* (*P* = 0.014), and *Selenomonas* (*P* = 0.050) at 12 months, as well as the genus *Porphyromona*s (*P* = 0.040) at 24 months; (**C**) At the species level, children in the TFV + OHE group demonstrated a higher abundance in *C. granulosa* than their peers in the OHE group at 12 months (*P* = 0.002, Kruskal-Wallis test, adjusted by Bonferroni correction for multiple tests). Note: G1: Group 1 (topical fluoride varnish, TFV); G2: Group 2 (oral health education, OHE); G3: Group 3 (TFV + OHE); *P-value less than 0.05; **P-value less than 0.01.

The community composition bar plot illustrated the Top 10 most abundant taxa at genus level, and the genus *Leptotrichia* was the most abundant taxon observed among the three groups during the whole study period (Fig. 3A). Among these, significant changes were observed in genus *Capnocytophaga* at both the 6- (*P* = 0.021) and 12-month assessments (*P* = 0.014). After the cessation of interventions, the 24-month findings revealed that the distribution of genus *Capnocytophaga* became similar among the three groups, while genus *Porphyromona*s differed across these groups (*P* = 0.040). Additionally, there were differences in the relative abundance of genus *Saccaribactetia_(TM7)_[G-1]* at 6 months (*P* = 0.034), as well as *Selenomonas* at 12 months (*P* = 0.050) across the three groups. At the species level, children who received TFV + OHE interventions showed a higher relative abundance in *Capnocytophaga granulosa* than those children who only received OHE intervention at 12 months (*P* = 0.002).

## DISCUSSION

To the best of our knowledge, this was the first 24-month three-armed, randomized controlled trial investigating the impact of TFV, OHE, and TFV + OHE on caries prevention as well as the oral microbiota among children with early mixed dentition.

The genus *Leptotrichia* was the most abundant taxon observed among the recruited children, consistent with another recent study (19). *Leptotrichia* are facultative anaerobic species predominantly detected in oral cavities and can act as opportunistic pathogens of human diseases (21). A case-control study conducted by Qudeimat et al. (22) reported that species belonging to *Leptotrichia* were significantly abundant among caries-active children. However, another matched case-control study exhibited a higher relative abundance of *Leptotrichia* among caries-free children than children with rampant caries (*P* = 0.023) (19). Furthermore, a study comparing the distribution patterns of oral microbiota among various caries layers revealed that *Leptotrichia* was one of the most abundant genera detected on the uncavitated tooth surface, while their abundance decreased in carious dentin and necrotic pulp tissues (23). As early pulp involvement is a common clinical feature observed among children with rampant caries, it is possible that *Leptotrichia* was less abundant among children with rampant caries. In our study, the severity of caries status on each tooth surface was assessed using ICDAS (17). Since the severity of caries was similar across the three groups throughout the whole study period, no significant changes in the abundance of *Leptotrichia* were observed.

During the intervention period, there were no statistical differences in Shannon diversity index, while significant differences were observed in the relative abundance of genus *Capnocytophaga*. Zhang et al. (24) also reported similar findings after fluoride application. However, the study conducted by Zhang and colleagues only observed the short-term effects of fluoride treatment on dental plaque microecology, as the 16S rRNA sequencing was conducted at baseline, 3 and 14 days, and they found that microbial community returned to original level after 2 weeks. Another study also demonstrated that short-term application of stannous fluoride (SnF2) gel showed limited impact on the alteration of microbial community (25). Comparing to the previous studies, this 24-month trial revealed a long-term impact of TFV and OHE on the composition of oral microbiota. At the species level, a lower abundance of *C. granulosa* was detected among children who did not receive fluoride. *Capnocytophaga* species are gram-negative, facultative anaerobic rods, requiring carbon dioxide for growth. There are nine species under the genus *Capnocytophaga*. Among those, *C. granulosa* and *Capnocytophaga haemolytica* have been detected in subgingival dental plaque samples collected from adults with chronic periodontitis (26). Capnocytophaga species were also found to be more prevalent in patients with dental calculus than those calculus-free individuals (27). A pre-and post-intervention study involving 52 children treated with TFV investigated the dynamic alterations in the microbial community structure, illustrating that the abundance of *C. granulosa* decreased significantly after 3 days but gradually returned to baseline levels by the 14th day after TFV (24). However, few long-term studies have investigated the impact of TFV on the alterations of *C. granulosa*. Although TFV might have the potential to influence the abundance of *C. granulosa*, the underlying mechanism remains unclear. Further studies are needed to explore the relationship between fluoride and *C. granulosa*.

Considering that cariogenic bacteria are likely to produce organic acids and survive in low-pH environments, the salivary pH values were assessed throughout the study period. A recent case-control study (19) reported that preschoolers with rampant caries exhibited significantly lower salivary pH values than caries-free children (6.93 ± 0.42 vs 7.18 ± 0.28, *P* < 0.01). Moreover, the salivary pH levels were positively correlated with the abundance of *Veillonella* and negatively correlated with *Cardiobacterium* (*P* < 0.01) (19). Although our primary findings revealed that the salivary pH values were statistically lower in the TFV group than the other groups after 12-month intervention, the mean difference between groups was only approximately 0.2 units, which might have limited clinical or ecological relevance. Therefore, the impact of salivary pH levels on the alteration of oral microbiota was not further explored in this study.

Regarding the clinical outcomes, the caries status of entire dentitions as well as the PFMs was evaluated. Considering the coexistence of primary and permanent teeth in the mixed dentition, DMFT/dmft scores were calculated (3). Additionally, “caries progression” was used to describe the scenarios where a carious lesion progressed to an advanced ICDAS code (11). No statistical differences were found in full-mouth caries experience among the three groups, which aligns with another 24-month randomized controlled trial, showing that biannual application of 5% NaF varnish did not show additional effects on caries prevention among children if OHE was provided (9). Anderson et al. (10) also found no significant differences in caries progression among children treated with semi-annual application of TFV or OHE and emphasized the importance of instructing parents about toothbrushing with fluoride toothpaste for caries prevention. In a three-armed clinical trial, 300 preschool children were randomly allocated into three groups, including the control group (without interventions), OHE group, and OHE + TFV group. The main findings suggested that both OHE or OHE + TFV were efficient in reducing caries incidence among young children, and parents’ oral health-related knowledge was improved by OHE interventions (28). Yawary and Hegde (29) also integrated OHE as a crucial component of the dental caries management protocol for pediatric patients. When comprehensive OHE was applied along with 38% silver diamine fluoride, 78% active caries were subsequently arrested at the 6-month follow-up. Moreover, this approach significantly improved the children’s oral health-related quality of life (29).

At the 12-month follow-up visit, the proportion of children with new carious lesions on PFMs was statistically higher in the TFV group (35.1%) than in the OHE group (16.3%). Upon adjusting for children’s demographics and family socioeconomic status, the binary logistic regression model revealed that children receiving TFV only were more likely to have new caries on their PFMs compared to those in the OHE group (OR = 2.86, 95% CI 1.33, 6.14), as well as those in the TFV + OHE group (OR = 2.28, 95% CI 1.09, 4.78). One possible reason might be the fact that in mixed dentition, the PFMs are vulnerable to occlusal caries rather than proximal caries due to the presence of narrow and deep fissures, incomplete post-eruptive maturation, poor oral hygiene status, and inappropriate oral hygiene habits (3, 30, 31). However, TFV is typically recommended for caries prevention in patients with active caries on smooth tooth surfaces rather than the occlusal surfaces (32). For children in the OHE group as well as the OHE + TFV group, they were instructed to brush teeth twice daily with fluoridated toothpaste, and the appropriate toothbrushing method was demonstrated on the OHE leaflets. By this way, these children had opportunities for fluoride exposure via toothbrushing and could also improve their toothbrushing techniques. Thus, better oral hygiene status was observed among children receiving OHE + TFV interventions than their peers receiving TFV alone after 12 months.

Moreover, there were no significant differences in caries incidence on PFMs between the OHE group and the TFV + OHE group after 24-month interventions. This finding contradicts another trial involving children with early mixed dentition (7), in which all the recruited children received OHE, and the test group received additional semi-annual TFV. The 24-month visit revealed that DFS increment and the caries incidence of the PFMs were significantly lower in the test group than the control group (7). One possible explanation might be the intervention frequencies and durations differ between the two studies. Our study involved a biannual application of TFV in the first 12 months, and a final examination was conducted at the 24-month visit to monitor whether the caries-prevention effect of the interventions can be sustained after the interventions ceased. However, the study conducted by Wang et al. (7) applied TFV four times over a 24-month follow-up period. Another contributing factor might be the strategy of OHE interventions. The OHE topics in our study were derived from previous studies, as there is evidence that these OHE topics positively influence children’ oral health-related behaviors (11, 33). Furthermore, the OHE interventions in our study were delivered in three parts: Part I was conducted at school, where investigators illustrated the key points of the OHE materials to the children individually in their classrooms. Part II took place at home, where children were instructed to take leaflets home and review and practice the content with their parents. This take-home learning and practice pattern could sustain the OHE information in their daily oral hygiene practices. Lastly (Part III), during follow-up visits, these topics were revisited to reinforce the children’s understanding of the OHE information. Therefore, the OHE information delivered to the recruited children would be less likely to fade away compared to a single OHE talk given to children at school.

The main limitation of this study was that the majority of the participants came from families with high socioeconomic status. Over half of the parents held a postgraduate degree, and more than 90% of those parents were both employed full time. Additionally, the recruited children showed better oral hygiene practices when compared to other studies conducted in the same province (3, 34). Specifically, over 87% children had prior dental visit experience, and approximately 84% children brushed their teeth at least twice daily at baseline. It is documented that OHE interventions can be influenced by family socioeconomic factors and parents’ oral health literacy (11, 35). The OHE interventions provided in this study might have yielded greater benefits for the recruited children, as their parents were more capable of interpreting and reinforcing the OHE information effectively. Conversely, there is evidence that fluoridation is tended to benefit children from low socio-economic status more than those from more affluent families, as the absolute fluoridation-related benefit for children form poorest families was a reduction of 2.0 mean decayed and filled primary tooth surface (dfs), while the corresponding benefit for their peers from high-income families was only 0.4 dfs per child (36). Although our main findings support that OHE is a promising strategy for caries prevention among children with early mixed dentition, these findings might not be generalized to children from low socio-economic backgrounds.

Additionally, due to the nature of OHE and TFV interventions, the participants were not blinded to the allocation concealment in this study. As human behaviors could be influenced by knowledge or beliefs, blinding or masking is vital to reduce such bias in a clinical trial (37). Since the caries prevention benefits of fluoride are well-known, if parents realized their children were assigned to the TFV group, they might assume their children’s oral health would improve with the intervention and may spend less time on daily oral health practices for their children. Moreover, although it is recommended that parental supervised toothbrushing should be maintained until the age of 8 (38), we did not investigate parental involvement in children’s daily toothbrushing practices. The DI-S score of the recruited children was lower at baseline but increased at the 6-month visit, followed by a gradual decrease at the 12- and 24-month visits. We hypothesized that since the children were younger at baseline, their parents were likely to supervise and assist them with toothbrushing. However, during the study period, parents might have assumed that their children, having participated in the oral health promotion programs, could brush their teeth independently. Consequently, parental involvement in daily toothbrushing might have diminished.

Furthermore, a study conducted among children with mixed dentition exhibited significant differences in the microbial structure of salivary and supragingival plaque samples (39). Lin et al. (23) demonstrated that the distribution patterns of oral microbial communities varied across different sites, including buccal enamel surface, superficial carious lesion, deep caries dentin, and necrotic pulp tissue. Salivary microbiota might be able to serve as a good proxy target for the total oral microbiome, as it is independent of different sites within the oral cavity. In our study, both saliva samples and supragingival dental plaque samples were collected from the participants. However, only dental plaques were analyzed using 16S rRNA sequencing, while the saliva samples were exclusively used for pH assessment. Given these limitations, more well-designed prospective studies are warranted to verify the impact of OHE and TFV on caries prevention.

### Conclusion

OHE, with or without additional TFV, could be recommended for dental caries prevention among children with early mixed dentition. Among the Top 10 most abundant genera, *Capnocytophaga*, *Saccaribactetia_(TM7)_[G-1]*, *Selenomonas*, and P*orphyromonas* differed across the three groups after interventions. Moreover, the 12-month assessment revealed a lower abundance of *C. granulosa* among children who did not receive TFV.

While fluoride application might potentially influence the composition of supragingival dental plaque, particularly affecting the abundance of *Capnocytophaga* species, the specific mechanisms and clinical implications remain unclear and warrant further investigation. Additionally, more well-designed randomized controlled trials involving children from diverse socioeconomic backgrounds are needed to explore optimal strategies for caries prevention.

## Data Availability

The sequence data have been deposited in SRA database (PRJNA1237516).

## SUPPLEMENTAL MATERIAL

Supplemental materials spectrum.00902-25-s0001.docx Additional experimental details and Table S1.

ASM does not own the copyrights to Supplemental Material that may be linked to, or accessed through, an article. The authors have granted ASM a non-exclusive, world-wide license to publish the Supplemental Material files. Please contact the corresponding author directly for reuse.
