# Supplementary material for: Impact of fluoride varnish and oral health education on caries increment and dental plaque microbiota among children with early mixed dentition: a three-armed 24-month randomized controlled trial
Source: Microbiol Spectr. 2025 Jul 28;13(9):e00902-25. doi: 10.1128/spectrum.00902-25 (PMC12403721; doi:10.1128/spectrum.00902-25)
Supplement: Supplemental materials — Additional experimental details and Table S1. [file spectrum.00902-25-s0001.docx]

**Supplemental materials**

***DNA extraction and amplification*** ***of the collected dental plaque samples***

The microbial DNA was extracted from 131 dental plaque samples using HiPure Stool DNA kit (Magen, China) following the manufacturer’s protocol. The 16S rRNA gene V3-V4 region was amplified using primers 341F (CCTACGGGNGGCWGCAG) and 806R (GGACTACHVGGGTATCTAAT)^[1,2]^. The polymerase chain reaction (PCR) was performed using reagents from New England Biolabs (USA), and the reaction system included 10μL 5x Q5® Reaction Buffer, 10 μL Q5® High GC Enhancer, 1.5 μL 2.5 mM dNTPs, 1.5μL Forward Primer (10μM), 1.5μL Reverse Primer (10μM), 0.2μL Q5® High-Fidelity DNA Polymerase, 50 ng of template DNA, and ddH_2_O to a final volume of 50 µL. The cycling conditions were listed as follows: initial denaturation at 95 ℃ for 5 min, followed by 30 cycles of 95 ℃ for 1 min, 60 ℃ for 1 min and 72 ℃ for 1 min, and final extension at 72 ℃ for 7 min.

***Purification and Illumina sequencing***

PCR products were assessed using 2% agarose gel electrophoresis, purified using AMPure XP Beads (Beckman, USA), and quantified using Qubit 3.0 Fluorometer (Thermo Scientific Inc., USA). Qualified libraries were sequenced on Novaseq 6000 Platform using PE250 mode by Gene Denovo Biotechnology Co., Ltd (Guangzhou, China).

***Quality control and clustering***

The yielded sequences were quality filtered using FASTP ^[3]^(v0.18.0). The clean reads were merged into tags using FLASH^[4]^ (v1.2.11). Additionally, the tags were further filtered using the recommended default settings proposed by Bokulich et al. (2013)^[5]^. The high-quality sequences were de-noised using DADA2 (v1.14.1) ^[6]^, and the amplicon sequence variants (ASVs) were generated accordingly. Furthermore, chimeric DNA sequences are likely to arise during PCR amplification, which can be misinterpreted if undetected. UCHIME was employed to identify and eliminate chimeras ^[7]^.

**Clinical parameters among children selected for microbiological study**

The salivary pH values, Simplified Debris Index (DI-S) and caries experiences of children who were randomly selected for microbiological analysis were summarized in Table S1. Children’s caries status was compared by Mann-Whitney test, while the other outcomes were analyzed by Student’s T tests. There were no significant differences in the caries status, oral hygiene status, nor salivary pH values between children whose dental plaque samples were analyzed and those whose samples were not analyzed.

**Table S1**. Comparison of clinical parameters between children whose dental plaque samples were analyzed and those whose plaque samples were not analyzed.

| Outcomes | Plaque analyzed | Plaque not analyzed | *p*-value |
| --- | --- | --- | --- |
| DMFS/dmfs |  |  |  |
| Baseline | 8.15(6.98) | 8.09(7.71) | 0.668 |
| 12 months | 13.30(10.27) | 12.47(9.93) | 0.621 |
| 24 months | 13.33(10.09) | 13.45(9.89) | 0.966 |
| DI-S |  |  |  |
| Baseline | 0.98(0.60) | 1.08(0.64) | 0.449 |
| 6 months | 1.48(0.55) | 1.41(0.57) | 0.697 |
| 12 months | 1.53(0.49) | 1.49(0.72) | 0.266 |
| 24 months | 1.42(0.72) | 1.39(0.72) | 0.581 |
| Salivary pH levels |  |  |  |
| Baseline | 7.44 (0.49) | 7.49 (0.42) | 0.086 |
| 6 months | 7.39 (0.25) | 7.36 (0.27) | 0.982 |
| 12 months | 7.14 (0.30） | 7.21(0.40) | 0.113 |
| 24 months | 7.04(0.31) | 7.12(0.32) | 0.692 |

**References**

1. Guo M, Wu F, Hao G, et al. Bacillus subtilis Improves Immunity and Disease Resistance in Rabbits. Front Immunol, 2017,8: 354.
2. Zhu J, Liu S, Wang H, et al. Microplastic particles alter wheat rhizosphere soil microbial community composition and function. J Hazard Mater, 2022,436: 129176.
3. Chen S, Zhou Y, Chen Y, et al. fastp: an ultra-fast all-in-one FASTQ preprocessor. Bioinformatics, 2018,34(17): i884-i890.
4. Magoc T, Salzberg S L. FLASH: fast length adjustment of short reads to improve genome assemblies. Bioinformatics, 2011,27(21): 2957-2963.
5. Bokulich N A, Subramanian S, Faith J J, et al. Quality-filtering vastly improves diversity estimates from Illumina amplicon sequencing. NAT METHODS, 2013,10(1): 57-59.
6. Callahan B J, Mcmurdie P J, Rosen M J, et al. DADA2: High-resolution sample inference from Illumina amplicon data. Nat Methods, 2016,13(7): 581-583.
7. Edgar R C, Haas B J, Clemente J C, et al. UCHIME improves sensitivity and speed of chimera detection. Bioinformatics, 2011,27(16): 2194-2200.
